# In-situ particle analysis with heterogeneous background: a machine learning approach

**DOI:** 10.1038/s41598-024-59558-7

**Published:** 2024-05-08

**Authors:** Adeeb Ibne Alam, Md Hafizur Rahman, Akhter Zia, Nate Lowry, Prabuddha Chakraborty, Md Rafiul Hassan, Bashir Khoda

**Affiliations:** 1 Department of Mechanical Engineering, University of Maine, Orono, ME 04469 United States; 2 Computer Science, University of Maine at Presque Isle, Presque Isle, ME 04769 USA; 3 Department of Electrical & Computer Engineering, University of Maine, Orono, ME 04473 USA

**Keywords:** Heterogeneous image, Particle detection with deep learning, Particle entrainment, Image processing, YOLO, Mechanical engineering, Characterization and analytical techniques

## Abstract

We propose a novel framework that combines state-of-the-art deep learning approaches with pre- and post-processing algorithms for particle detection in complex/heterogeneous backgrounds common in the manufacturing domain. Traditional methods, like size analyzers and those based on dilution, image processing, or deep learning, typically excel with homogeneous backgrounds. Yet, they often fall short in accurately detecting particles against the intricate and varied backgrounds characteristic of heterogeneous particle–substrate (HPS) interfaces in manufacturing. To address this, we've developed a flexible framework designed to detect particles in diverse environments and input types. Our modular framework hinges on model selection and AI-guided particle detection as its core, with preprocessing and postprocessing as integral components, creating a four-step process. This system is versatile, allowing for various preprocessing, AI model selections, and post-processing strategies. We demonstrate this with an entrainment-based particle delivery method, transferring various particles onto substrates that mimic the HPS interface. By altering particle and substrate properties (e.g., material type, size, roughness, shape) and process parameters (e.g., capillary number) during particle entrainment, we capture images under different ambient lighting conditions, introducing a range of HPS background complexities. In the preprocessing phase, we apply image enhancement and sharpening techniques to improve detection accuracy. Specifically, image enhancement adjusts the dynamic range and histogram, while sharpening increases contrast by combining the high pass filter output with the base image. We introduce an image classifier model (based on the type of heterogeneity), employing Transfer Learning with MobileNet as a Model Selector, to identify the most appropriate AI model (i.e., YOLO model) for analyzing each specific image, thereby enhancing detection accuracy across particle–substrate variations. Following image classification based on heterogeneity, the relevant YOLO model is employed for particle identification, with a distinct YOLO model generated for each heterogeneity type, improving overall classification performance. In the post-processing phase, domain knowledge is used to minimize false positives. Our analysis indicates that the AI-guided framework maintains consistent precision and recall across various HPS conditions, with the harmonic mean of these metrics comparable to those of individual AI model outcomes. This tool shows potential for advancing in-situ process monitoring across multiple manufacturing operations, including high-density powder-based 3D printing, powder metallurgy, extreme environment coatings, particle categorization, and semiconductor manufacturing.

## Introduction

Powder particles and granular materials are important forms of material commonly used in manufacturing parts such as additive manufacturing^1^ and powder metallurgy^2^, transforming surfaces for rust protection^3–5^, controlling roughness and conductivity^6^, creating meta surface^7,8^, enabling self-cleaning hydrophilic surfaces^9,10^, enhancing properties like viscosity modifications^11^. Particle-size composition plays a key role for the functionality as well as performance^12^ of the manufactured part and coating properties. The characteristics of suspensions, emulsions, and mixtures often used in powder-based manufacturing depend largely on powder materials size, shape and distribution of powder materials. These factors control the porosity and mechanical properties of sintered^13^ and additively manufactured samples^14^, improves flowability and wettability of yield stress fluids^15^, and influence thermal and electrical conductivity^16^. Therefore, characterizing the particle size distribution before and during manufacturing becomes important at advanced production and scientific facilities^17–19^.

**Figure 1:**
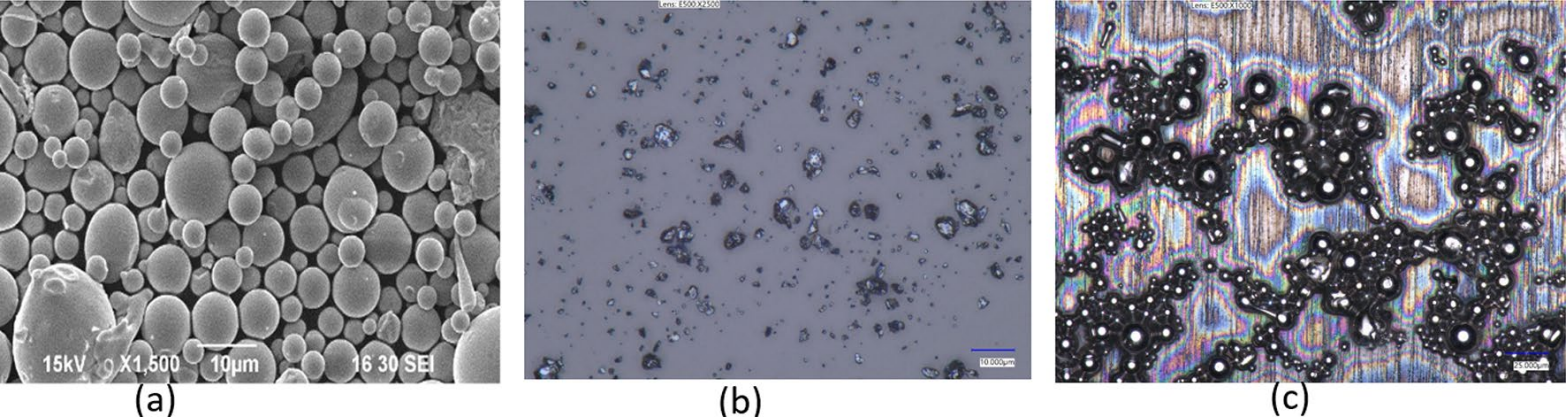
Particle images at different environment (**a**) highly controlled SEM image of NicroBraz particles only (low noise) (**b**) molybdenum particles on substrate as glass slide (medium background distraction) (**c**) polymer entrapped NicroBraz particles on tungsten rod substrate (heterogeneous interface).

Among many powder manufacturing processes, melt atomization processes are widely used in industries due to their better control in shape and distribution of particles. However, polydispersity is the intrinsic characteristic of the particle produced and often requires classification and separation. For bulk particles, their size and distribution are often measured with particle size analyzer (i.e., laser diffraction technique) during the preprocessing stage of their applications at a controlled environment as shown in Fig. 1a. This bulk particle analysis technique requires some degree of dilution and may compromise measurement accuracy^20^. Particle size analysis is also performed on static or dynamic images by different image analysis techniques. Static image analysis is performed when the particles are stationary and motionless whereas dynamic image analysis is carried out in a media of gas or liquid with particles and matrix^21^. The image-based particle analysis techniques are effective in the presence of distinguishable morphologies (i.e., polydispersity, color, and shapes). Static images can be obtained by optical, confocal, scanning electron microscopy (SEM) or Transmission electron microscopy (TEM) techniques. The quantitative analysis of SEM or optical images is important to understand the powder characteristics for its variety of applications. Visual and structural information including particle size range, size distribution shape and morphology obtained from images is crucial since it provides technical and scientific insights to the process of material synthesis, fabrication, and manufacturing operations^22^.

Most image analysis tools drive researchers to extract information from the microscopic and spectroscopic images without prepossessing the particles (e.g., adding media). However, these tools are often user specific, involve semantic knowledge, and produce qualitative results. A widely used, conventional image processing tool, applied for general purposes is ImageJ developed by National Institute of Health (NIH)^23^. Kumara et al. performed image analysis of gravels (2–19 mm) through ImageJ software^24^. They captured the 2D images of gravels in a transparent sheet as background using a digital single-lens reflex (DSLR) camera. Berardi et al. studied the size expansion of tablets during disintegration for pharmaceutical applications^25^. They also captured images of tablets with a DSLR camera and performed image analysis through ImageJ to determine projected area and aspect ratio. Lee et al.^26^ analyzed the confocal laser scanning microscopy (CLSM) images of pellet coating using ImageJ and calculated the coating thickness from the irregular shape measurement of pellets. He et al.^27^ studied the influence of process parameters including solution concentration, collection distance, voltage and collection speed on the diameter and orientation of nanofibers made of electrospinning process. They investigated the surface morphology of nanofibers using SEM images and analyzing it through ImageJ. Depending upon the sample and imaging conditions, this software sometimes emits particles and incorrectly identifies particle boundaries which requires labor intensive post-processing.

Apart from ImageJ software, several automatic and semiautomatic software packages have also been used to determine particle size distribution. Mondini et al.^28^ developed a custom software named Pebbles to measure the surface morphology and diameter of nanoparticles. Phromsuwan et al.^29^ analyzed the nanoparticle size distribution in transmission electron microscopy (TEM) images using an automated image processing technique called Otsu binarization. Laramy et al.^30^ also developed particle analysis software with a customized algorithm and the MATLAB image analysis toolbox to detect the structure of nanoparticles in SEM images.

With the recent development in machine vision and deep learning process, researchers have reported automated image processing techniques to identify the surface morphology and segment regions of optical or spectroscopy images. Xu et al.^31^ identified microstructure properties in SEM images using machine learning (ML) techniques. Modarres et al.^32^ introduced convolutional neural network (CNN)- based models to extract surface morphological properties from SEM images. Massarelli et al.^33^ analyzed images from digital cameras and used computer vision and ML algorithms to count and classify the dimensional and morphological properties of microplastics in water. The morphology of core–shell and rod nanostructures is determined from SEM images using a combination of computer vision and ML techniques, available as a GUI software package^22^. The developed ML algorithms work efficiently with distinguishable background, controlled illumination, and high-resolution images. Thus, the background substrate containing the particles is often chosen to ensure contrast, which helps these algorithms in extracting a clear particle outline. This analysis is feasible when particles are in a bulk state, meaning they are analyzed as raw material before **their applications, in a controlled or semi-controlled environment (pre-processing stage), as shown in Fig. 1a,b.

**Figure 2:**
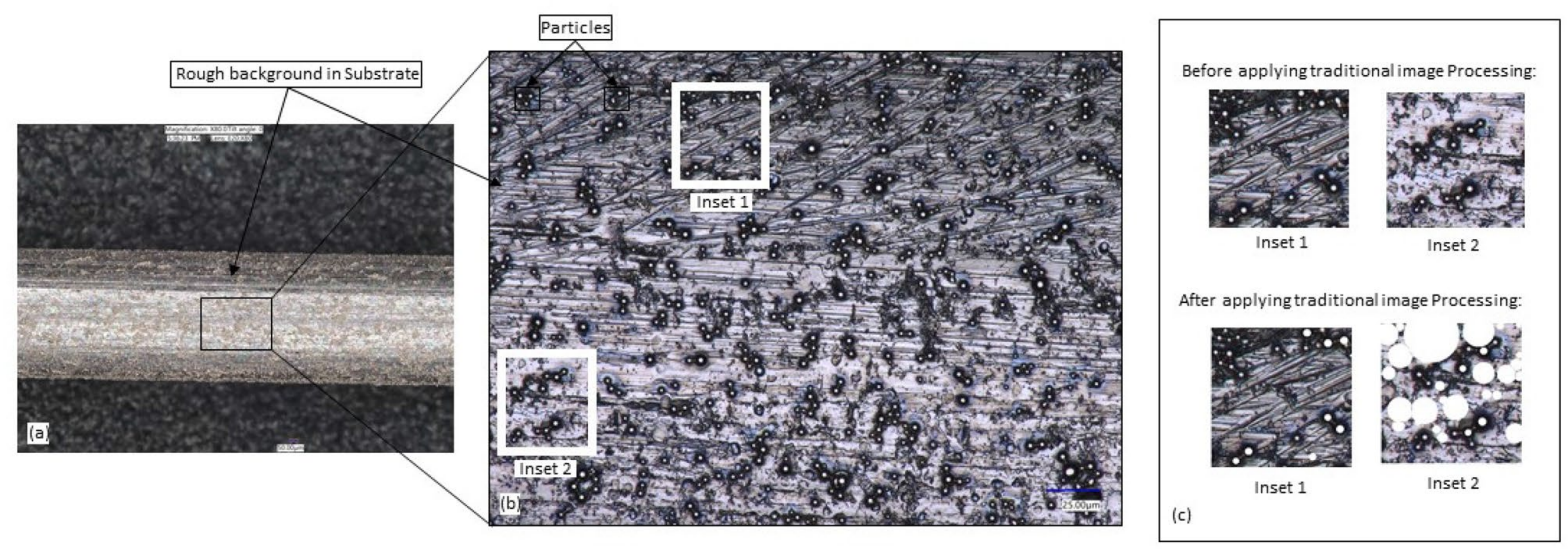
Conventional image processing algorithms applied to a heterogeneous image taken by VHX 7000 digital 4 K microscope after dip coating process. (**a**) Shows the particle–substrate system with 80X Zoom (**b**) with 1000X zoom, two insets show two different regions of the same image. (**c**) shows the effect of traditional image processing methods applied to the two inset images, which generates completely different outcomes for the same input.

However, when particles are at the intermediate state of a manufacturing process (work-in-process), image background noise can arise from particle, substrate and process trio, as shown in Fig. 1c. Analyzing particles at such a heterogenous interface can be challenging due to the lack of contrast, high image noise, uneven illumination, and hazy backgrounds, as shown in Fig. 2. For example, in our recent work we developed a poly-disperse inorganic particle sorting process by entrapment onto a cylindrical rod^34^. We used Ni-based brazing powder (Nicrobraz 51; Wall Colmonoy company, Ohio; Spherical dia range ~ 0–100 µm) as bulk particles. The bulk particles are sieved with Gilson Performer III shaker through Stainless Steel 635 Mesh (20 µm) to reduce the polydispersity and are analyzed using SEM (Avg. 5.69 μm) before the sorting process. In our developed particle sorting process, the spherical micro-particles are added into a liquid carrier system (LCS) with binder polymer. A thin metal rod is dipped into the mixture, and particles of different sizes are entrained on the rod’s surface due to the balance between viscous drag, and capillary action. Entrainment based sorting of bi-dispersed ceramic particles is also proposed by Sauret et al.^35^ where a flat glass substrate is used for dipping and entrained glass particles are analyzed for size distribution. The entrained or entrapped particle on the substrate often needs to be characterized in-situ before they can be sent to the next step of the manufacturing or coating process. For example, in our recent work, we used the entrained particles on an elastic rod to control the friction coefficient^36,37^. By analyzing the entrained particles, we can predict and hence control the friction coefficient for various applications including robot grippers, knot design, wire entanglement etc.

The entrained particles on metal rods have been used to develop a novel porous structure manufacturing technique with rod reported in our earlier work^38,39^. The characteristics of the entrained particles on the substrate define the diffusion bonding strength and thus their size and distribution are important to analyze before they are placed in a vacuum furnace. However, the particle–substrate-process trio creates a heterogeneous interface as a background (shown in Fig. 1), which is often difficult to analyze with conventional image processing techniques. Due to the deep drawing fabrication of the metal rod, a rough and irregular surface morphology is common, which contributes further towards the heterogeneity of the image. As a result, the pixel contrast between particle and substrate is difficult to differentiate in such a heterogeneous surface. Similar complexity can occur for in-situ measurement of particles in other manufacturing processes (e.g., brazing, sintering, refractory material coating, spray painting etc.). In such circumstances, most existing tools (e.g., ImageJ, variable thresholding) generate significant errors in particle analysis without labor intensive semantic knowledge (compared in the result section). For example, as shown in Fig. 1, due to localized variation, a single image processing parameter cannot detect particles across the entire region.

In this work, we have:

(i) Dealt with the challenge of in-situ particle analysis from complex and varied backgrounds typical of heterogeneous particle–substrate (HPS) interfaces in the manufacturing industry, which is scarce in literature.
(ii) Defined and demonstrated heterogeneous particle–substrate (HPS) interface as complex background, which are common in powder-based manufacturing processes.
(iii) Implemented and tested traditional particle detection methods, such as those using size analyzers and based on dilution, image processing, or deep learning that are popular for particle identification from homogeneous background and demonstrate their struggle to accurately detect particles against the HPS interfaces.
(iv) Proposed and developed a novel framework by combining a state of art deep learning with image pre- and post-processing to address the aforementioned challenge.
(v) Proposed and developed a set of novel post processing algorithms for enhancing the efficacy of the particle detection framework.
(vi) Conducted extensive experimentation to evaluate the efficacy of the proposed framework.

## Background and motivation

Conventional image processing algorithms can automatically identify and count particles in an image and can measure their size, distribution, surface coverage. However, these algorithms have significant drawbacks when applied to counting particles, especially in the heterogeneous backgrounds discussed earlier. Moreover, the performance of the image processing techniques is highly dependent on several parameters, including image quality, light source, background surface texture, optimal threshold value, and particle morphology.

**Figure 3:**
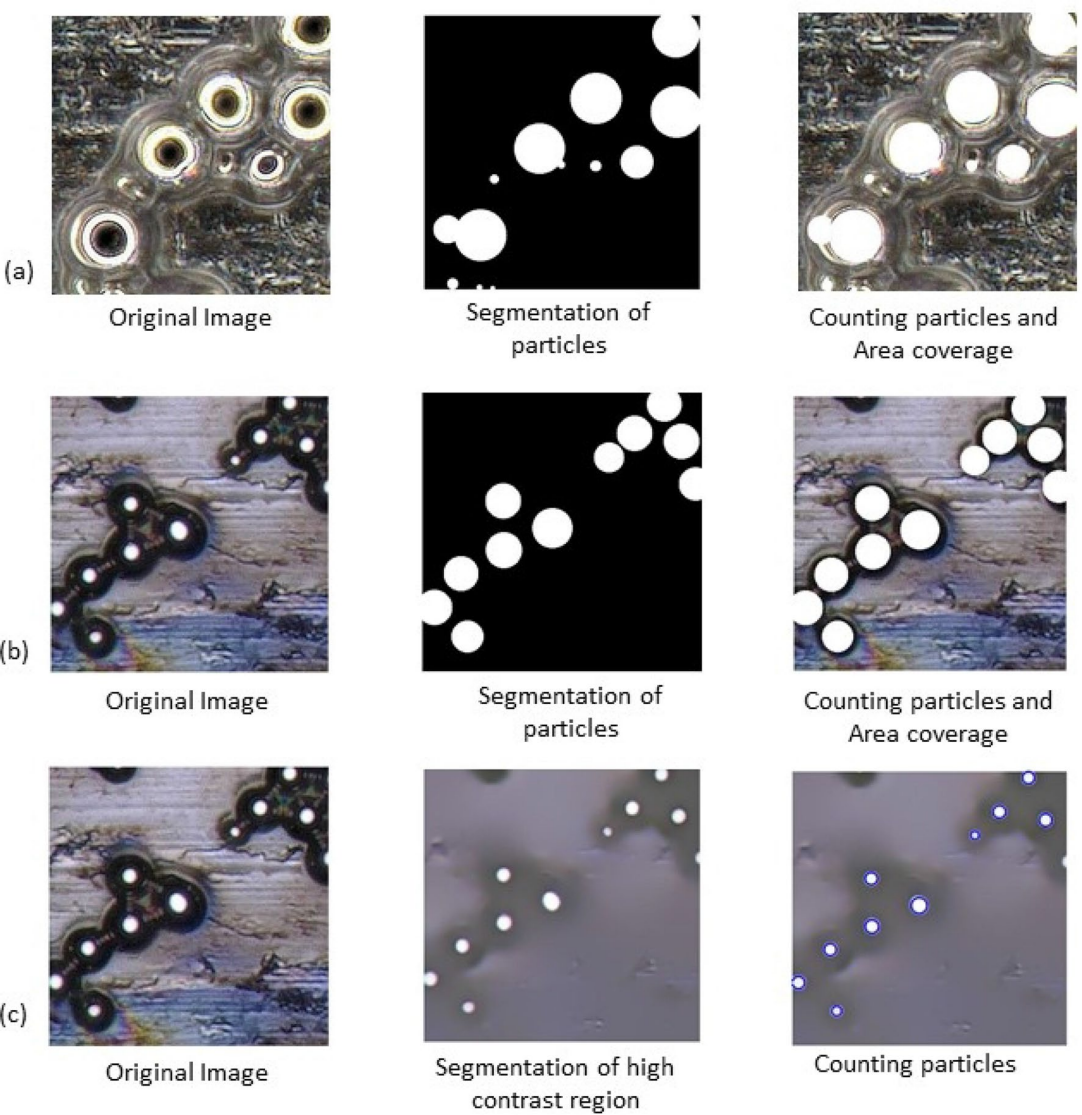
Demonstration inconsistency in particle identification outcome in HPS images with different existing algorithm (**a**) Image taken with full circle illumination and custom thresholding algorithm to segment the particle coverage area, (**b**) Image taken with coaxial full ring illumination segmentation is done by changing parameters of the customized thresholding algorithm, (**c**) Image taken with coaxial full ring illumination and localized contrasting algorithm is applied.

For example, the heterogeneous images in Fig. 3 illustrate the performance of image processing under varying illumination conditions. The image presented in Fig. 3a utilizes full ring illumination to transform the gray-scale image into a black and white image. Then the watershed algorithm along with the morphological operations (dilation and opening) are applied to identify the regions covered by individual particles. An empirical threshold value, such as Otsu’s thresholding, can be selected when other variable thresholding methods^40,41^ appear not suitable for the problem, as shown in Fig. 3. This can be a common attribute of heterogeneous background discussed earlier. Figure 3b,c show the particle identification performance using an image processing approach on the images with coaxial full ring lighting. As mentioned, the figures demonstrate that the particle morphology has completely changed due to variation in lighting conditions; a careful analysis of these images reveal that each particle can be identified by the central illuminated circle rather than the light ring. For the image shown in Fig. 3c, a comparable performance has been achieved by localized contrasting algorithms. These results prove that there are no universal image processing parameters available that can be applied to heterogeneous images for particle characterization from images. The performance of the traditional image processing approach varies with optimal choice of the threshold value along with the variation of background noise and lighting illumination. Thereby, an automated computational tool would assist both professionals and novice users in automatically characterizing particles from heterogeneous images. This approach will eliminate the need for manual parameter selection in image processing and facilitate the capture of semantic knowledge, thereby improving the performance of the particle identification technique.

To illustrate the current challenge, we evaluated the performance of ImageJ software with the assistance of a graduate student who has at least 40 h of experience using the software. When implemented on the HPS datasets, the results, as shown in Table 1, indicate that recall is always higher than precision which can be interpreted as over detection due to the presence of heterogeneity. Additionally, the prediction of surface area showed significant deviation from the ground truth, attributed to the software's inability to differentiate between particle clusters and nuances on the substrate, a common issue in the manufacturing domain. Expertise in image processing is crucial for identifying particles in images with heterogeneous backgrounds. Correct particle identification may require a deep understanding of filtering, edge detection, image conversion, and more. However, particle identification via image processing alone may not always yield optimal results due to the necessity of adjusting various parameters like threshold values and skewness degrees for each image which may require immense knowledge on filtering, edge detection, image conversion and many other approaches. This research introduces a semi-supervised innovative approach, reducing the need for deep technical knowledge by combining basic image processing with advanced deep learning, enabling more accessible particle identification from heterogeneous images without requiring detailed knowledge of image processing or artificial intelligence.

**Table 1:** Performance metrics of ImageJ software with HPS dataset.

Dataset	Recall	Precision	F1	TP	FP	FN	Particle Surface Area (sq. pixel)${(\times 10}^{3})$	
							Ground Truth	Predicted
A	0.857	0.857	0.857	12	2	2	37.4	240.6
B	0.923	0.800	0.857	12	3	1	45.7	315.2
C	0.944	0.739	0.829	17	6	1	30.2	197.3
D	0.889	0.667	0.762	24	12	3	44.9	82.2
E	0.941	0.800	0.865	16	4	1	22.7	156.8

## Sample preparation and image capturing

**Figure 4:**
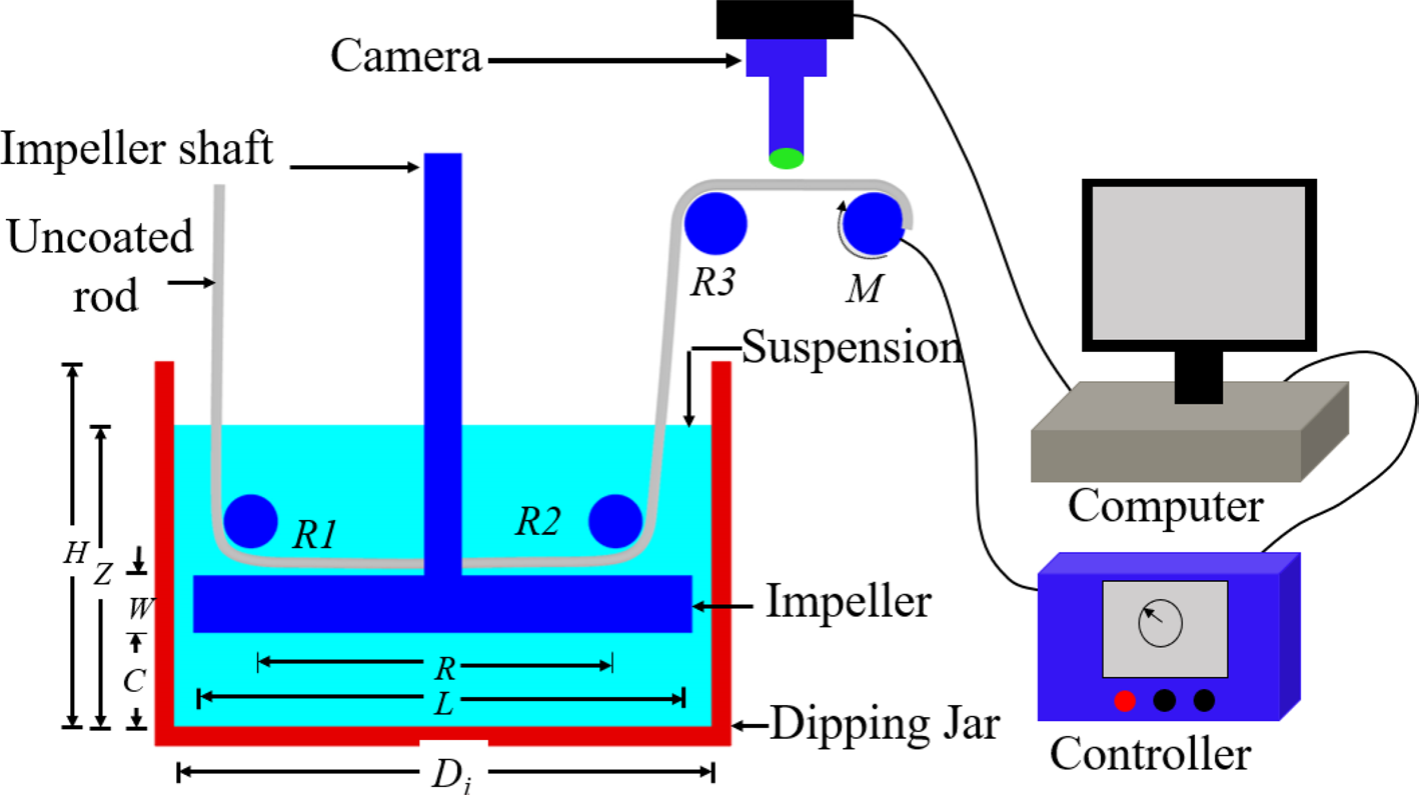
Schematic of continuous dipping process for particle entrainment and imaging setup.

We utilized an in-house continuous dipping system to produce heterogeneous images, simulating a continuous manufacturing process by entraining particles onto a substrate. Initially, we formulated a polymer solution using Polymethyl Methacrylate (PMMA) and the solvent 1,3- Dioxolane, both sourced from Sigma Aldrich. After stirring for 8 h, a clear and uniform liquid carrier solution (LCS) is obtained. We then introduced particles into the LCS to formulate the dipping mixture, ensuring the ratio of polymer and solvent (LCS) to particles maintains the mixture within the Newtonian regime. We chose cylindrical rods as substrates, and these are dipped through the mixture for particle entrainment, as illustrated in Fig. 4. To prevent particle sedimentation during the dipping process, we agitate the mixture to disperse the particles uniformly, forming a 'pseudo-suspension'^42,43^.

As the rod is withdrawn from the mixture, a balance between the viscous drag and capillary action facilitates the entrainment of particles onto the rod substrate. This balance, characterized by a dimensionless capillary number, dictates the dipping characteristics and the subsequent particle transfer. Details of this are further discussed in our previous work^42^. Once entrained, the particles adhere to the substrate, using the binder as a glue, and the solvent rapidly evaporates. An in-situ analysis of the transferred particles can be conducted using an in-line microscope or camera, as depicted in Fig. 4. The resulting surface of the rod, dotted with particles, serves as a representation of the particle–substrate-process system, showcasing a heterogeneous morphology.

For framework evaluation, we constructed five different types of heterogeneous images by varying the trio of particle, substrate, and imaging processes. The samples are created from our laboratory developed continuous entrainment process by dipping and images are taken afterward. By adjusting the particle morphologies (i.e., type, size, and shapes), substrate and capillary number during the entrainment process, we've captured images at various ambient lighting which creates a diverse level of heterogeneous background (Sample A ~ E in Table 1). These images were captured with a VHX 7000 digital 4 K microscope (by KEYENCE Corporation Ltd., IL) at 1000X magnification, covering a 300 × 200 micron area. Following this, our proposed particle characterization framework was applied to these varied heterogeneous images for both training and validation purposes.

## Design and Implementation of the Proposed AI Framework

**Figure 5:**
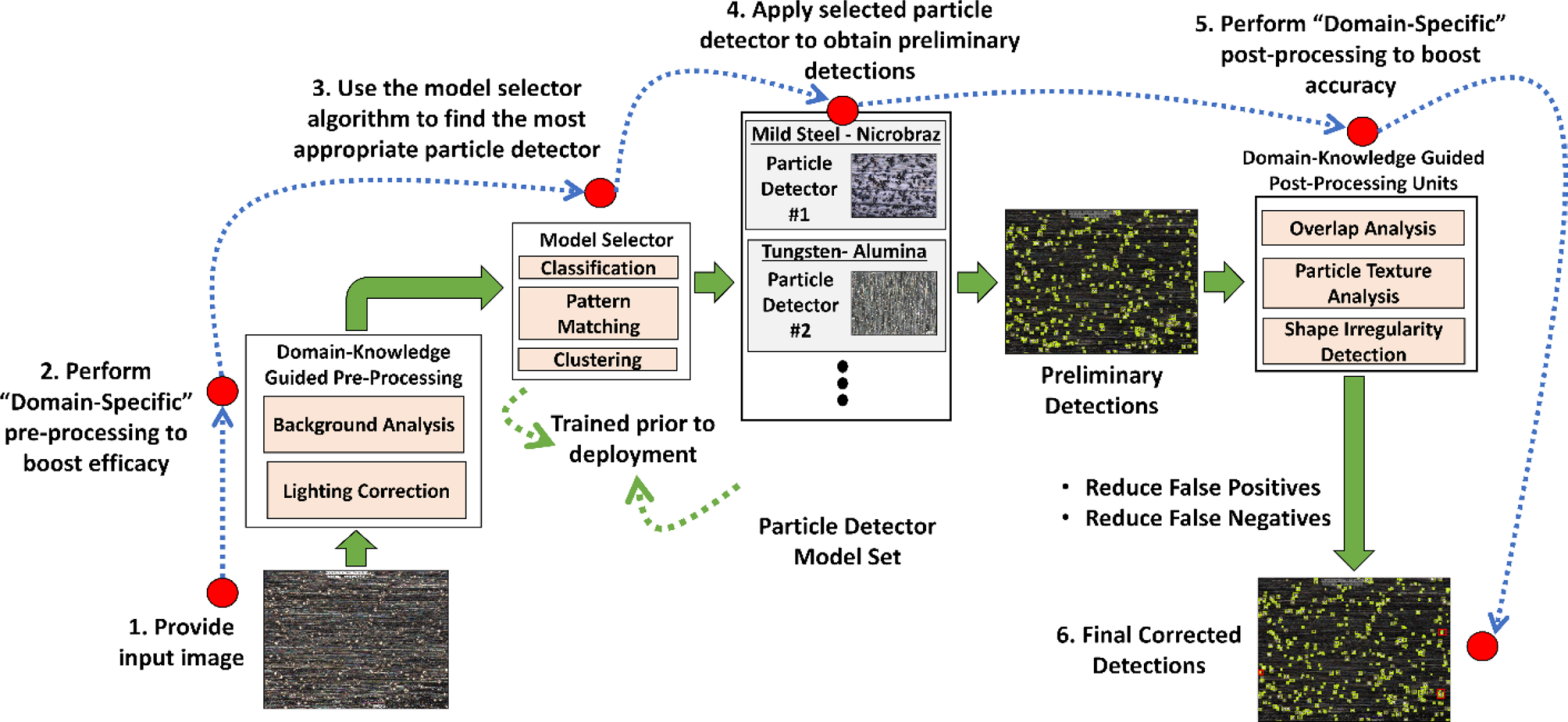
Overview of the proposed modular particle detection framework.

In this section we describe the proposed particle detection framework. As shown in Fig. 5, this framework has four main steps: preprocessing, model selection, AI-Guided particle detection, and post processing.

### Preprocessing strategies

**Figure 6:**
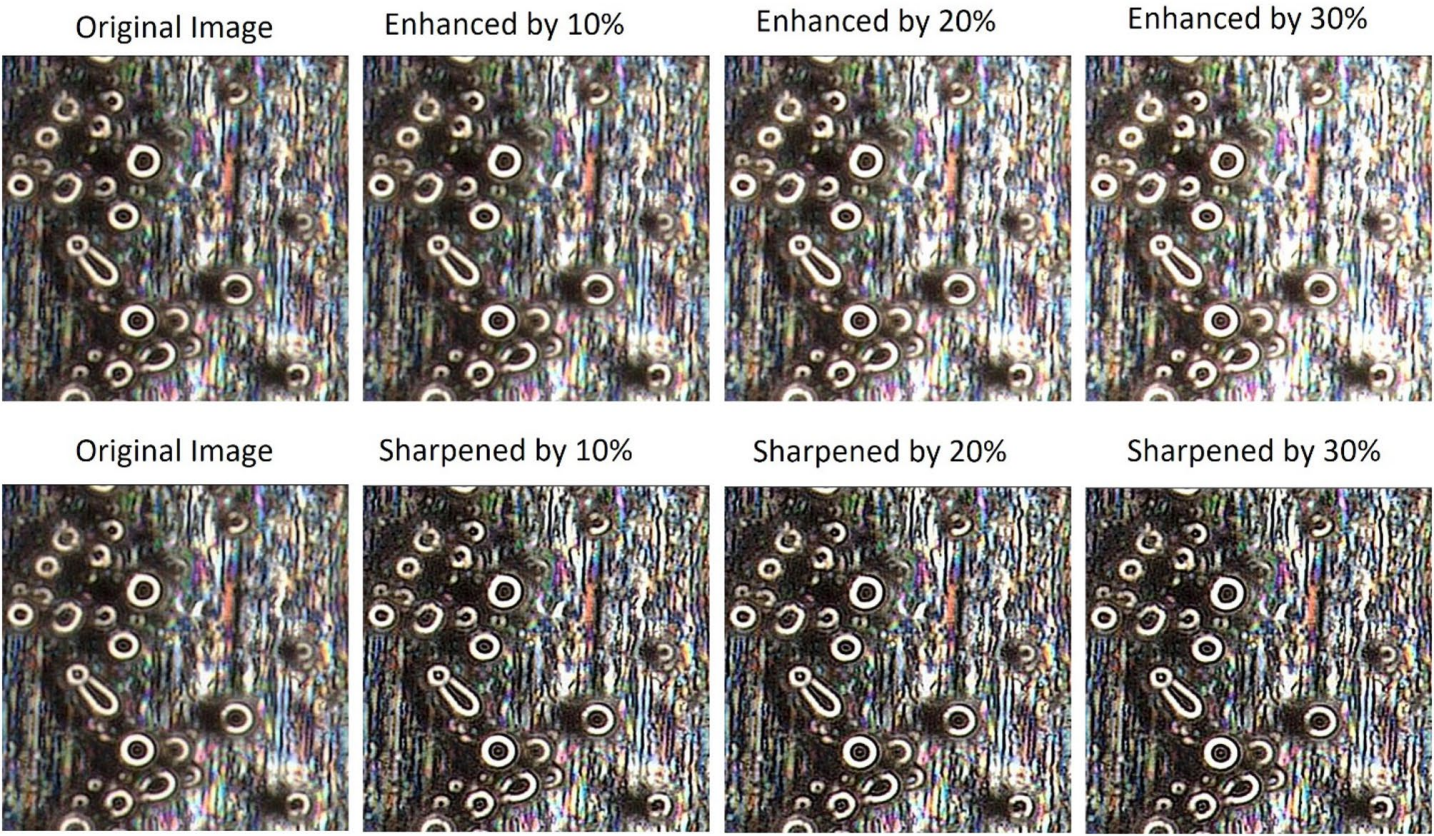
Preprocessing effects: Sharpening leads to better definition of smaller particles while enhancing visibility.

The provided particle–substrate image requires some preprocessing before it is ready for the AI model. In this study, we experimented with enhancement and sharpening. Image enhancement increases the dynamic range of gray levels through linear stretching, expands dark pixel values, performs gamma correction, and equalizes the intensity histogram. Image sharpening is typically carried out by adding a high pass filter output of the image with the base image. This increases contrast, making object detection and localization more accurate. We demonstrated the visual effects of these pre-processing strategies on a sample image in Fig. 6. Sharpening indeed makes detection easier for smaller objects and enhancement makes dimmer objects brighter, thereby facilitating their detection in subsequent steps. Together, these techniques reduce noise impact, enhance device capabilities, and contribute to cleaner data for analysis. Overall, they improve image visualization and help to make more accurate insights and decisions. The framework is designed in a modular fashion allowing future addition of more preprocessing units such as background removal and other image correction techniques. The effectiveness of this process is investigated in the results section.

### Model selection

Extensive quantitative analysis on the HPS images has shown that separate AI detectors are necessary to accurately detect particles for each particle–substrate combination. In the presence of a set of AI-models, we must determine, for an incoming image, which AI model should be used for it. To address this, our study employs an image classifier model, effectively guiding the selection process by identifying the most suitable AI model to be employed for the analysis of a specific image. We use Transfer Learning (TL) as a Model Selector (MS) where MobileNet acts as a base model to categorize the particle–substrate dataset. While we chose MobileNet for its increased inference time efficiency, this model can be replaced with one that is either more accurate, at the cost of speed, or faster, at the cost of accuracy. The model selector is designed to enable end-to-end automation by eliminating any human-interaction that would generally be required for determining the right model for a given particle–substrate combination.

To train the MS model we first split up the five different particle–substrate datasets into training, validation and testing with a ratio of 65%, 15% and 20% respectively where each dataset acted as labels. We trained the model up to 50 epochs with a batch size of 64, using Adam optimizer^44^ with an initial learning rate of 0.001. We also used early stopping where training was terminated if validation loss did not improve for 10 successive epochs. Our model selector achieved 99.69% testing accuracy and 0.9967 F1-score.

### AI-guided particle detection

Once a specific AI particle detector has been selected for an input image, we utilize it for detecting bounding boxes for each image particle. The proposed framework is designed to leverage diverse object detection techniques depending on which one serves the purpose best. These techniques include DetectorNet, SPPNet, MSC Multibox, YOLO, SSD, MASK RCNN, and RetinaNet. Each technique has its own unique strengths and weaknesses. The selection of a specific algorithm (or a set) will depend on priorities placed on metric such as: (1) Inferencing speed; (2) Accuracy; (3) Model size; (4) Training speed; (5) Data requirements. For example, SSD is faster than YOLO but has difficulty detecting smaller objects. Mask RCNN can be accurate but lacks inferencing speed.

**Figure 7:**
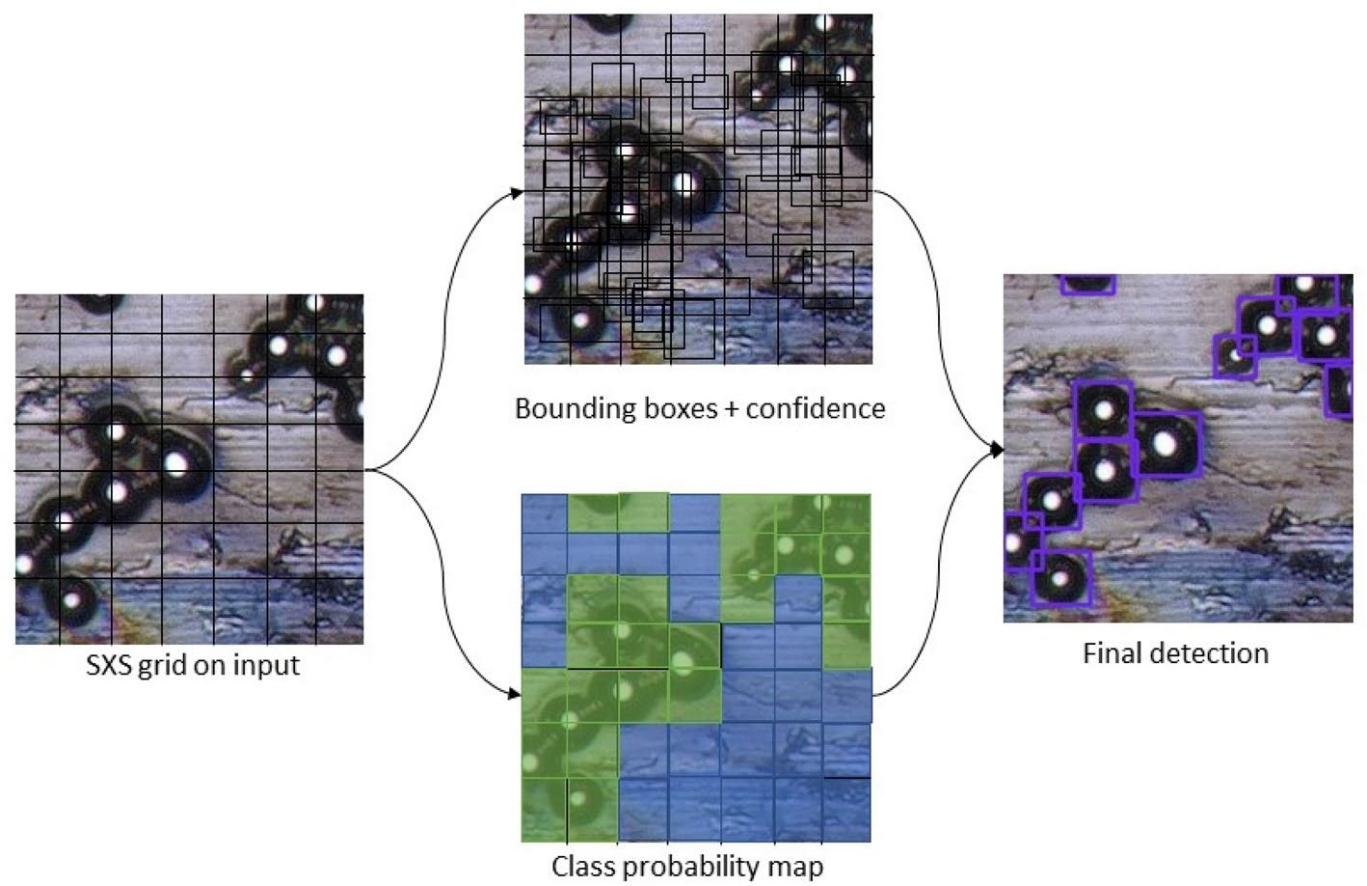
Object detection strategy for YOLO net.

Most of these particle detection algorithms have certain similarities. For example, in the case of the YOLO algorithm, the image is divided into a set of grids/cells followed by a CNN based detection of the presence or absence of an object (Fig. 6). The scores (indicating the presence or absence of an object) for each of the grids are considered as confidence corresponding to the respective grids. If the confidence score for a grid is high enough to have an object (particle) in it, the corresponding grid is expanded further to identify a suitable bounding box. Once the bounding box with the highest score is achieved this score is considered as class probability for the corresponding bounding box. The same approach is repeated for each grid of the input image. Thereby, each bounding box has confidence as well as class probability. Confidence refers to whether the box contains any object or not and the class probability refers to the probability of the class of the object that is inside the bounding box. Finally, the class label of each bounding box is determined following the class probability map as shown in the right most figure of Fig. 7. The overall framework is highly flexible and can incorporate any set of object detection algorithms. However, for our experiments we have decided to move ahead with YOLO due to its demonstrated ability in detecting small objects (our use case). We have picked YOLOv5 for our experiments due to its ability to learn from a small dataset (an unavoidable limitation for this particle detection domain). Also, YOLOv5 can handle images of variable size which would be useful during inferencing. CSPDarknet53 (Conv, BatchNorm, SiLU, C3, and SPPF) acts as the backbone for the YOLOv5 model. Next YOLOv5 up-samples the output feature map to create more complex features and the head portion of the model is responsible for making the final predictions.

Apart from the AI algorithm selection, we have also observed that training separate AI-models for all possible particle–substrate-image combinations yields the best results. Hence, a set of AI models will be trained and stored for performing this step (the selection of which is done using the prior step). The select AI model will output a set of bounding boxes indicating the location of each particle in the input image.

### Domain knowledge guided post processing

**Figure 8:**
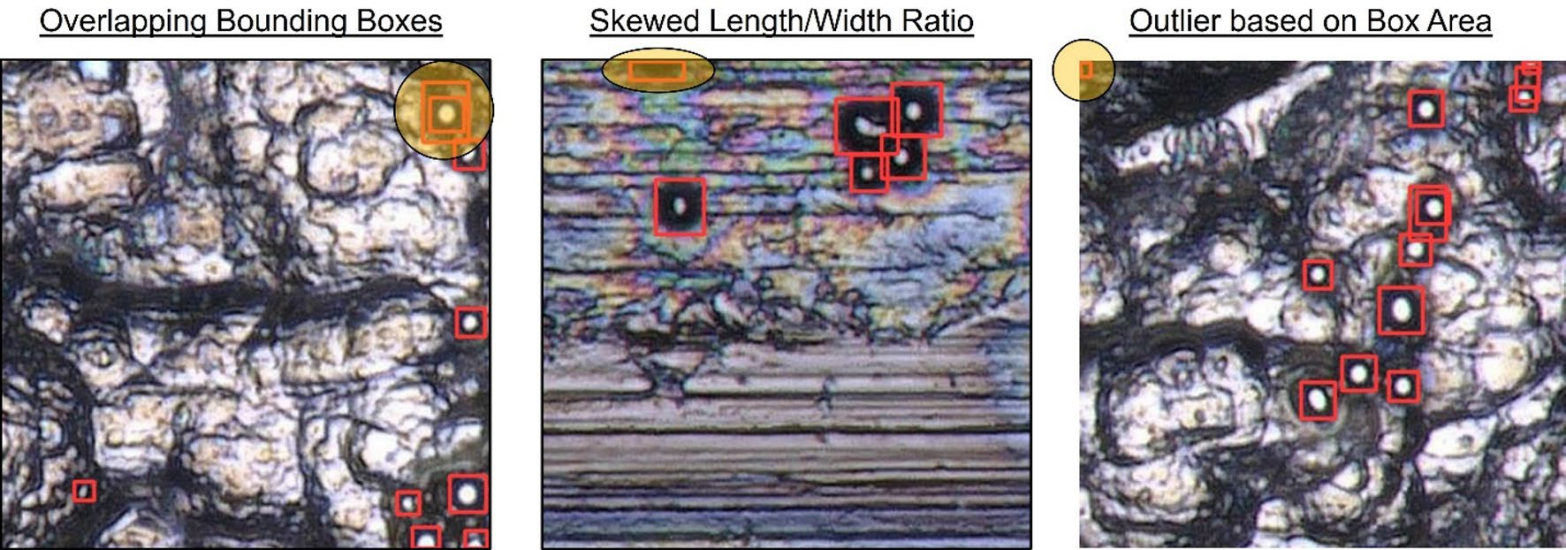
Common detection issues observed.

The detections obtained from using the AI models can be extremely noisy, reducing the overall efficacy of the framework. The biggest concern arises due to having a large number of false positives (detections that are wrong). Specifically, these false positives can be due to overlapping detection boxes, phantom detection, and double counting (see Fig. 8). We propose three different algorithms to remove some of these false positives, thereby increasing the overall efficacy of the framework.

**Figure 9:**
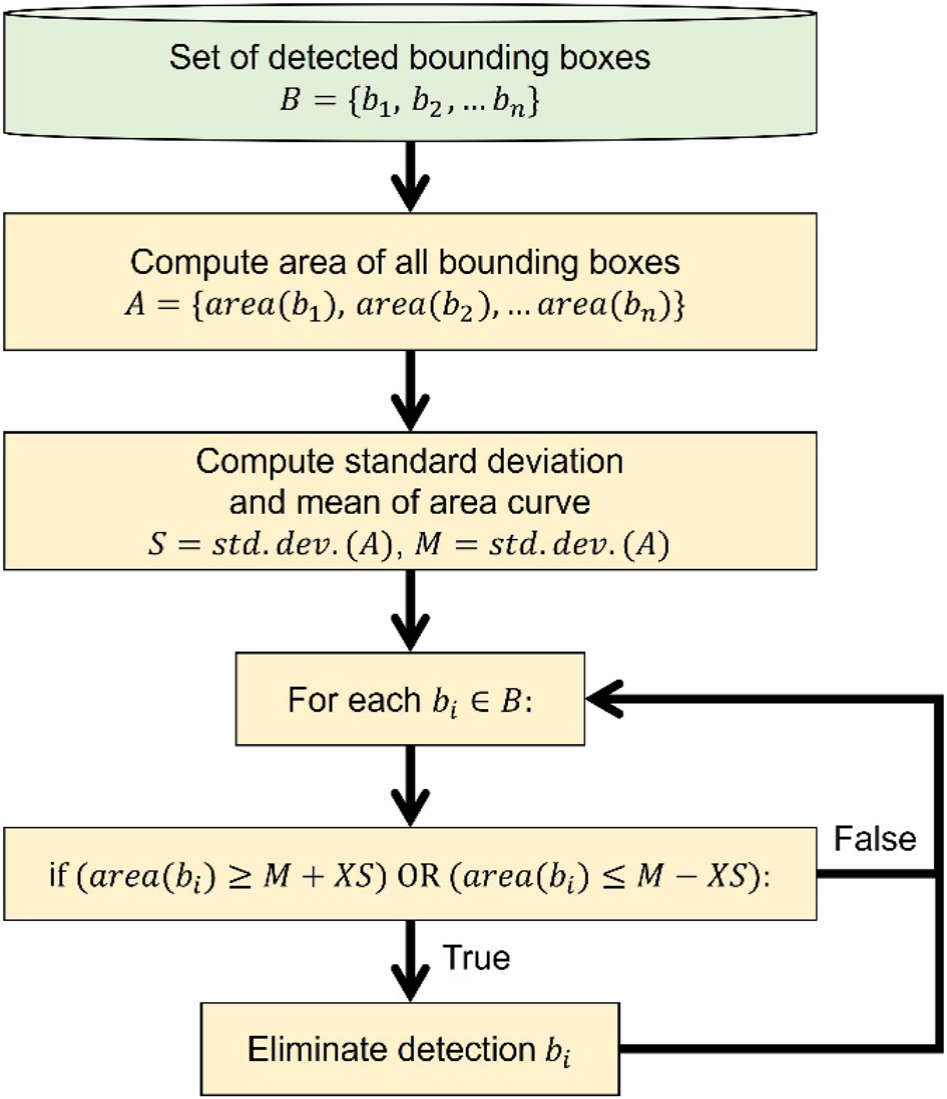
Algorithm for pruning based on area.

**Figure 10:**
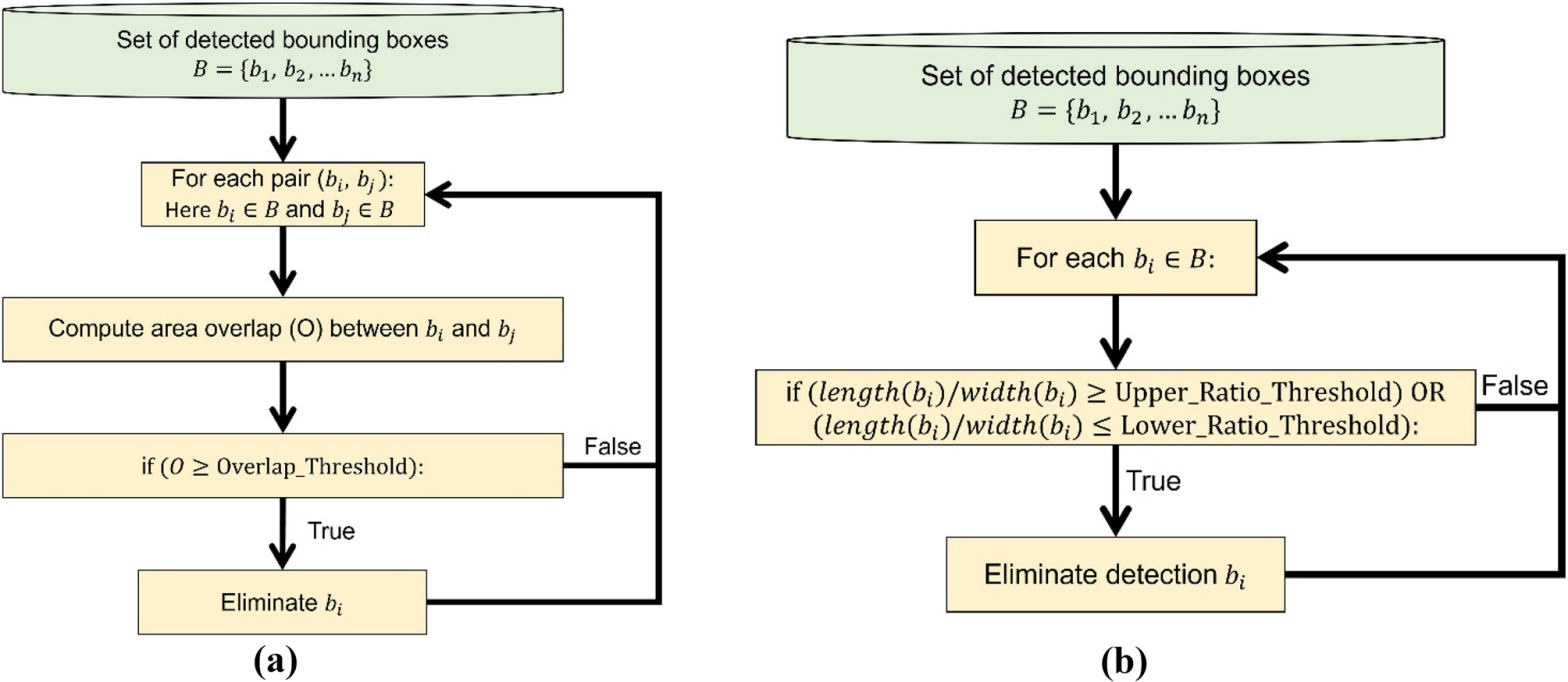
From left to right: (**a**) algorithm for pruning overlapping boxes; (**b**) algorithm for pruning boxes with skewed shape.

In Fig. 9, we describe the first proposed algorithm used to detect bounding boxes that are extremely small or extremely large. This process involves computing the area of the current set of bounding boxes, calculating the mean and standard deviation of the distribution, and then eliminating any bounding box located X standard deviation away. Here X can be provided as a user input and determined based on empirical analysis. In Fig. 10a, we describe the process of pruning overlapping bounding boxes. This process involves calculating the overlap for each pair of bounding boxes based on the number of pixels in the intersecting area, then removing one of the boxes if the overlap exceeds a certain user-defined threshold. In Fig. 10b, we describe the process of pruning bounding boxes with skewed shapes compared to other detections. This process involves calculating the ratio between the length and the breadth of each bounding box and then eliminating all detections beyond user-defined upper and lower bounds, as determined by empirical observations. We opt for post-processing because it is an effective and computationally efficient method to enhance prediction accuracy and precision by leveraging domain knowledge in particle detection strategies.

## Results and validation

### Experimental setup

In this study, we used a desktop computer with Ubuntu 22.04.03 operating system, Intel Core i9 2.5 GHz 16 Cores CPU, NVIDIA GeForce RTX 3060 12 GB GPU, 2 TB HDD, and 32 GB RAM. We utilized the PyTorch deep learning framework (version 2.0.1) with CUDA support for accelerated training. We consider five different types of particle–substrate-process image dataset (discussed in Table 2) where each image has resolution of 2880 × 2160. The original dataset comprised 12 images each for Samples A, B, and C, 13 images for Sample D, and 8 images for Sample E. With the help of web-based annotation software “Roboflow” (app.roboflow.com), we divided the original dataset into training, validation, and test sets with ratios of 70%, 15%, and 15% for data Sample A, B, and C and with a ratio of 60%, 15% and 25% for data Sample D and E. After splitting, we got 8 training, 2 testing and 2 validation images for Sample A, B, and C where for D we got 8 training, 3 testing and 2 validation images and for E we got 5 training, 2 testing and 1 validation images. We then tiled each image into a 6 × 5 block and resized each block to 416 × 416 resolution. After tiling images, we got 240 training, 60 testing and 60 validation images for Sample A, B, and C and for D we got 240 training, 90 testing and 60 validation images and for E we got 150 training, 60 testing and 30 validation images. We augmented the training dataset with random clockwise and anticlockwise rotation, and random vertical and horizontal flips. After random augmentation we got 660, 720, 720, 720, and 450 training samples for Sample A, B, C, D and E.

**Table 2:** Sample preparation with varying particles, substrates, and processes for generating heterogeneous images.

Sample	Particle	Substrate and Roughness value	Imaging	Sample Image
A	Nicrobraz LM, Spherical Shape	AISI 1006 Mild Steel Rod ${(R}_{a}=0.26\mathrm{ \mu m})$	Full Ring lighting	
B	Nicrobraz LM, Spherical Shape	AISI 1006 Mild Steel Rod ${(R}_{a}=0.26\mathrm{ \mu m})$	Co-axial lighting	
C	Nicrobraz LM, Spherical Shape	AISI 306 Stainless Steel Rod ${(R}_{a}=0.37\mathrm{ \mu m})$	Co-axial lighting	
D	Alumina, Irregular Shape	Tungsten (W) Rod ${(R}_{a}=0.19\mathrm{ \mu m})$	Full Ring lighting	
E	Nicrobraz LM, Spherical Shape	Titanium Rod used in TIG welding ${(R}_{a}=2.14\mathrm{ \mu m})$	Full Ring lighting	

We used YOLOv5 for predicting particle bounding boxes. We utilized the small-sized variant of YOLOv5 (YOLOv5s) with a model configuration of 416 × 416 input resolution. We adopted a batch size of 16 and employed the SGD optimizer with an initial learning rate of 0.01, momentum of 0.937 and weight-decay of 0.005. We also used early stopping where the model stops its training process if the validation loss does not improve for 10 successive epochs. The model was trained for a total of 100 epochs.

The table above shows the experimental results of five different particle detection datasets using YOLOv5s. To better understand the contributions and interactions of individual components within our framework, we have performed several ablation studies and report them in Table 3. Particularly, we performed analyses under four different conditions: (1) without preprocessing and post-processing, (2) without preprocessing but with post-processing, (3) with preprocessing but without post-processing, and (4) with both preprocessing and post-processing. We utilized three metrics-recall, precision, and F1-score- to evaluate the performance of each model configuration and compare it with the overall framework performance as combined. These metrics can be calculated using the following mathematical expressions-

**Table 3:** Results with model selection.

Dataset	Pre-processing	Post-processing	Recall	Precision	F1	TP	FP	FN	Particle Surface Area (sq. pixel)${(\times 10}^{6})$	
									Ground Truth	Predicted
E	Without	Without	0.9325	0.8106	0.8673	428	100	31	25.6	28.2
	10%		0.9302	0.8609	0.8942	427	69	32		27.0
	20%		0.902	0.8961	0.899	414	48	45		25.1
	30%		0.9063	0.8927	0.8995	416	50	43		25.3
	Without	With	0.9325	0.8121	0.8682	428	99	31		28.2
	10%		0.9281	0.8623	0.894	426	68	33		26.8
	20%		0.902	0.8961	0.899	414	48	45		25.1
	30%		0.9063	0.8985	0.9024	416	47	43		25.0
C	Without	Without	0.915	0.9601	0.937	409	17	38	22.5	22.7
	10%		0.9146	0.9713	0.9421	407	12	38		21.9
	20%		0.9083	0.9713	0.9387	406	12	41		22.2
	30%		0.9105	0.9645	0.9367	407	15	40		22.5
	Without	With	0.9083	0.9598	0.9333	406	17	41		22.5
	10%		0.9034	0.9757	0.9352	402	10	43		21.6
	20%		0.8993	0.9734	0.9349	402	11	45		21.8
	30%		0.9016	0.9641	0.9318	403	15	44		22.4
B	Without	Without	0.8876	0.9497	0.9176	774	41	98	44.6	42.5
	10%		0.8842	0.9345	0.9087	771	54	101		42.6
	20%		0.8968	0.931	0.9136	782	58	90		43.6
	30%		0.8876	0.9337	0.9101	774	55	98		43.0
	Without	With	0.8624	0.9555	0.9066	752	35	120		40.7
	10%		0.8658	0.9426	0.9026	755	46	117		41.1
	20%		0.8739	0.9407	0.9061	762	48	110		41.6
	30%		0.8693	0.9404	0.9035	758	48	114		41.6
A	Without	Without	0.8989	0.8911	0.895	409	50	46	22.2	21.9
	10%		0.9077	0.9137	0.9107	413	39	42		21.9
	20%		0.8901	0.8804	0.8852	405	55	50		21.9
	30%		0.8835	0.9371	0.9095	402	27	53		20.9
	Without	With	0.8747	0.8964	0.8854	398	46	57		21.6
	10%		0.8835	0.9178	0.9003	402	36	53		21.4
	20%		0.8725	0.8822	0.8773	397	53	58		21.4
	30%		0.8593	0.9444	0.8999	391	23	64		20.2
D	Without	Without	0.8131	0.8452	0.8288	770	171	177	49.7	49.2
	10%		0.7855	0.8626	0.8222	747	119	204		47.2
	20%		0.7793	0.8817	0.8274	738	99	209		45.5
	30%		0.7926	0.8596	0.8248	753	123	197		47.9
	Without	With	0.794	0.8487	0.8205	752	134	195		47.8
	10%		0.7761	0.8719	0.8212	735	108	212		46.2
	20%		0.7614	0.8868	0.8193	721	92	226		44.2
	30%		0.7684	0.866	0.8143	730	113	220		45.9
Combine	Without	Without	0.8654	0.921	0.8924	2753	236	428	164.6	157.5
	10%		0.8699	0.9165	0.8926	2767	252	414		158.9
	20%		0.8658	0.9131	0.8888	2754	262	427		159.7
	30%		0.8655	0.9086	0.8865	2753	277	428		158.6
	Without	With	0.8475	0.9268	0.8556	2697	213	484		152.8
	10%		0.8444	0.9214	0.8812	2686	229	495		152.4
	20%		0.846	0.9184	0.8807	2691	239	484		154.7
	30%		0.8463	0.9122	0.878	2692	259	489		154.1

1 $$Recall = \frac{TP}{(TP+FN)}$$

2 $$Precision = \frac{TP}{(TP+FP)}$$

3 $$F1-Score = \frac{2*Recall *Precision}{Recall+Precision}$$

Where, TP (True positives) refers to correctly predicted boxes as particles, 'FP' (False Positives) refers to incorrectly predicted boxes as particles, and 'FN' (False Negatives) refers to particles that the model failed to identify. In the 'E' dataset, the YOLOv5 model achieved a recall of 0.9063, precision of 0.8985, and F1-score of 0.9024, following a training regimen that includes 30% image enhancement and sharpening (preprocessing) combined with postprocessing. In the 'E' dataset alone, the recall is higher than precision, suggesting that the model misses a significant number of particles, leaving them undetected. This can be attributed to the higher heterogeneity in substrate as shown in the roughness measurement. In this scenario, pre-processing steps help increase the precision which can be seen in the results. For the remaining four datasets (A–D), precision surpasses recall, indicating the beneficial impact of the postprocessing algorithm following the deep learning model's predictions. This improvement can be observed in the result table above. The “C” dataset model shows *recall of 0.9146, precision of 0.9713 and F1-score of 0.9421* in the combination of 10% image enhancement and sharpening without post-processing. Implementing the proposed framework, rather than relying on individual AI models, ensures consistency in both precision and recall, with their harmonic mean (F1-score) comparable to the outcomes of individual AI models. This indicates that the transfer learning-based model selection algorithm was able to classify the incoming image and pair it to the appropriate algorithm designed for the dataset. The proposed framework is also extremely scalable in terms of speed due to lightweight pre/post processing and decision-making models. Experimental results show 80.86 frames per second processing efficiency during inferencing.

## Conclusions

Particle size assessment is essential in metrology research due to the significant influence of particle size and its distribution on chemical, physical, and metallurgical activities. During the manufacturing process, particles merge with substrates or are suspended in liquid mediums, complicating their identification with conventional tools. In this paper, we presented a novel framework by combining state of art deep learning approaches with pre- and post-processing algorithms for particle detection in complex/heterogeneous backgrounds which are common in the manufacturing domain. The proposed frame detects and counts particles with very high accuracy, even in complex backgrounds with diverse morphology. Our comprehensive analysis reveals that within the proposed unified framework, precision and recall remain consistent across various HPS conditions, and their harmonic mean is comparable to outcomes from individual AI models.

The proposed framework can be adapted for in-situ measurement of particle size, distribution, and coverage, and thereby evaluate the quality of particles during manufacturing. The framework can be applied online to correct the process parameters during manufacturing due to its quick, immediate, and accurate outcome. This innovative tool holds promise for enhancing in-situ process monitoring for multiple manufacturing operations, such as high-density powder-based 3D printing, powder metallurgy, extreme environment refractory coatings, particle categorization and semiconductor manufacturing. Additionally, this algorithm holds the potential to reverse-engineer manufacturing characteristics, such as liquid carrier systems, benefiting various industrial applications, including blood diagnosis and wastewater treatment etc.

## References

[1] Cordova L (2017). Powder characterization and optimization for additive manufacturing. Tribol. Int.

[2] Samal P, Newkirk J (2015). Powder metallurgy methods and applications. ASM Handb. Powd. Metall..

[3] Coan T, Barroso GS, Motz G, Bolzán A, Machado RAF (2013). Preparation of PMMA/hBN composite coatings for metal surface protection. Mater. Res..

[4] Mariello M (2019). Reliability of protective coatings for flexible piezoelectric transducers in aqueous environments. Micromachines.

[5] Liu W, Li J, Huang X, Bi J (2021). Corrosion protection of Q235 steel using epoxy coatings loaded with calcium carbonate microparticles modified by sodium lignosulfonate in simulated concrete pore solutions. Materials.

[6] Holtzer, M., & Kmita, A. Mold and core sands in metalcasting: chemistry and ecology. In *Sustainable Development* (2020).

[7] Baumeister, P. *Optical Coating Technology* (SPIE Press, 2004).

[8] Stewart JW, Akselrod GM, Smith DR, Mikkelsen MH (2017). Toward multispectral imaging with colloidal metasurface pixels. Adv. Mater..

[9] Köstlin H, Frank G, Hebbinghaus G, Auding H, Denissen K (1997). Optical filters on linear halogen-lamps prepared by dip-coating. J. Non-crystal. Solids.

[10] Abubakar AA, Yilbas BS, Al-Qahtani H, Alzaydi A, Alhelou S (2020). Environmental dust repelling from hydrophobic and hydrophilic surfaces under vibrational excitation. Sci. Rep..

[11] Khoda B, Ahsan AMMN, Shovon SMN (2021). Dip coating from density mismatching mixture. J. Micro and Nano-Manufact..

[12] Gerasimov AM, Eremina OV, Cherkasova MV, Dmitriev SV (2021). Application of particle-size analysis in various industries. J. Phys.: Conf. Ser..

[13] Chávez-Vásconez R (2021). Effect of the processing parameters on the porosity and mechanical behavior of titanium samples with bimodal microstructure produced via hot pressing. Materials.

[14] Slotwinski JA, Garboczi EJ, Hebenstreit KM (2014). Porosity measurements and analysis for metal additive manufacturing process control. J. Res. Natl. Inst. Stand. Technol..

[15] Han X, Ghoroi C, To D, Chen Y, Davé R (2011). Simultaneous micronization and surface modification for improvement of flow and dissolution of drug particles. Int. J. Pharm..

[16] Murawski D, Behrens H (2017). Effect of particle size and pretreatment on the conductivity of glass powder during compaction. Zeitschrift für Physikalische Chemie.

[17] Zhang Y, Hu C, Wang X, Wang M, Jiang Z, Li J (2017). An improved method of laser particle size analysis and its applications in identification of lacustrine tempestite and beach bar: An example from the Dongying depression. J. Earth Sci..

[18] Syvitski, J. P. M. *et al*. *Principles, Methods, and Application of Particle Size Analysis* (Cambridge University Press Cambridge, 1991).

[19] Barishnikov, A. M. & Gaft, M. L. *The Application of Laser Element Online Analyzer MAYA for Extraction of Mineral Raw Materials and for Stabilization of Raw Mixtures in Nonferrous Metal Production* (2014).

[20] Black DL, McQuay MQ, Bonin MP (1996). Laser-based techniques for particle-size measurement: A review of sizing methods and their industrial applications. Progress Energy Combust Sci..

[21] Farkas D, Madarász L, Nagy ZK, Antal I, Kállai-Szabó N (2021). Image analysis: A versatile tool in the manufacturing and quality control of pharmaceutical dosage forms. Pharmaceutics.

[22] Kim H, Han J, Han TY-J (2020). Machine vision-driven automatic recognition of particle size and morphology in SEM images. Nanoscale.

[23] Underwood SJ, Gorham JM (2017). Challenges and approaches for particle size analysis on micrographs of nanoparticles loaded onto textile surfaces. NIST Spec. Publ..

[24] Kumara GHAJJ, Hayano K, Ogiwara K (2012). Image analysis techniques on evaluation of particle size distribution of gravel. GEOMATE J..

[25] Berardi A, Bisharat L, Blaibleh A, Pavoni L, Cespi M (2018). A simple and inexpensive image analysis technique to study the effect of disintegrants concentration and diluents type on disintegration. J. Pharm. Sci..

[26] Lee M-J (2011). In line NIR quantification of film thickness on pharmaceutical pellets during a fluid bed coating process. Int. J. Pharm..

[27] He H, Wang Y, Farkas B, Nagy ZK, Molnar K (2020). Analysis and prediction of the diameter and orientation of AC electrospun nanofibers by response surface methodology. Mater. Des..

[28] Mondini S, Ferretti AM, Puglisi A, Ponti A (2012). PEBBLES and PEBBLEJUGGLER: Software for accurate, unbiased, and fast measurement and analysis of nanoparticle morphology from transmission electron microscopy (TEM) micrographs. Nanoscale.

[29] Phromsuwan U, Sirisathitkul C, Sirisathitkul Y, Uyyanonvara B, Muneesawang P (2013). Application of image processing to determine size distribution of magnetic nanoparticles. J. Magn..

[30] Laramy CR, Brown KA, O’Brien MN, Mirkin CA (2015). High-throughput, algorithmic determination of nanoparticle structure from electron microscopy images. ACS Nano.

[31] Xu H, Liu R, Choudhary A, Chen W (2015). A machine learning-based design representation method for designing heterogeneous microstructures. J. Mech. Des..

[32] Modarres MH, Aversa R, Cozzini S, Ciancio R, Leto A, Brandino GP (2017). Neural network for nanoscience scanning electron microscope image recognition. Sci. Rep..

[33] Massarelli C, Campanale C, Uricchio VF (2021). A handy open-source application based on computer vision and machine learning algorithms to count and classify microplastics. Water.

[34] Khalil I, Khoda B (2022). Sorting of poly-disperse particle by entrapment using liquid carrier system. J. Manuf. Sci. Eng..

[35] Sauret A, Gans A, Colnet B, Saingier G, Bazant MZ, Dressaire E (2019). Capillary filtering of particles during dip coating. Phys. Rev. Fluids.

[36] Ibrahim Khalil M, Tong D, Wang G, Khalid Jawed M, Khoda B (2022). Systematic variation of friction of rods. J. Appl. Mech..

[37] Khalil MI, Islam MA, Tong D, Jawed MK, Khoda B (2024). Controlling surface of rods with entrained particle as asperities. J. Micro- and Nano-Manuf..

[38] Khoda B, Ahsan AMMN (2021). A novel rapid manufacturing process for metal lattice structure. 3D Print. Addit. Manuf..

[39] Khoda B, Ahsan AMMN, Shovon AN, Alam AI (2021). 3D metal lattice structure manufacturing with continuous rods. Sci. Rep..

[40] Otsu N (1979). A threshold selection method from gray-level histograms. IEEE Trans. Syst. Man Cybern..

[41] Wellner, P. D. Adaptive thresholding for the DigitalDesk. *Xerox, EPC1993–110,* 1–19 (1993).

[42] Shovon SM, Alam A, Gramlich W, Khoda B (2022). Micro-particle entrainment from density mismatched liquid carrier system. Sci. Rep..

[43] Khoda B, Gramlich W, Shovon SMN, Khalil I (2023). Effect of molecular weight on polymer solution facilitated transfer of non-Brownian particles. Progress Org. Coat..

[44] Kingma, D. P., & Ba, J. *Adam: A Method for Stochastic Optimization* (2014).

